# Lung cancer metastasis to the breast in patients with breast implants—first reports in the literature

**DOI:** 10.1093/jscr/rjaf598

**Published:** 2025-08-20

**Authors:** Chloe Jordan, Sarah Al-Hamali, Krzysztof Sosnowski, Laura Henderson, Susanna Polotto, Areeg Abbas, Georgette Oni

**Affiliations:** Addenbrookes Hospital, Cambridge University Hospitals NHS Foundation Trust, Hills Road, Cambridge, CB2 0QQ, United Kingdom; School of Medicine, University of Nottingham, East Block, Lenton, Nottingham NG7 2UH, United Kingdom; School of Clinical Medicine, University of Cambridge, Cambridge Biomedical Campus, Cambridge, CB2 0SP, United Kingdom; Addenbrookes Hospital, Cambridge University Hospitals NHS Foundation Trust, Hills Road, Cambridge, CB2 0QQ, United Kingdom; School of Medicine, University of Nottingham, East Block, Lenton, Nottingham NG7 2UH, United Kingdom; Sheffield Teaching Hospitals NHS Foundation Trust, Sheffield, S10 2JF, United Kingdom; Department of Histopathology, Nottingham University Hospitals, NHS Trust, Nottingham NG5 1PB, United Kingdom; Addenbrookes Hospital, Cambridge University Hospitals NHS Foundation Trust, Hills Road, Cambridge, CB2 0QQ, United Kingdom; School of Medicine, University of Nottingham, East Block, Lenton, Nottingham NG7 2UH, United Kingdom

**Keywords:** lung cancer, breast metastasis, breast implants, reconstructive surgery

## Abstract

The increasing use of breast implants in cosmetic and reconstructive surgery has brought attention to rare malignancies linked to the capsule that forms around them, such as breast implant-associated anaplastic large cell lymphoma, angiosarcomas and breast implant associated-squamous cell carcinoma. While breast metastasis from primary lung cancer is rare, it has not been previously reported in patients with breast implants. This case series presents two unique cases. The first involves a 46-year-old woman with left breast implants presenting with breast and axillary lumps and was diagnosed with contralateral small cell lung carcinoma . The second case features a 73-year-old woman with prior bilateral breast cancer and implant reconstruction, who presented with right breast swelling and was diagnosed with subsequent metastatic squamous cell carcinoma of the lung. Metastatic disease to the breast from a lung primary in patients with breast implants remains a rare and unreported phenomenon. This case series contributes to the literature by presenting two unique cases of metastatic lung cancer in patients with breast implants, both of whom presented primarily with breast-related symptoms. The chronic inflammation caused by breast implants may create a conducive environment for tumour growth and dissemination. Further research is critical for identification of pathogenesis and its prevention in future cases.

## Introduction

The use of breast implants is rising year on year due to increases in aesthetic and reconstructive breast surgery. In 2023 over 17 000 implant procedures were performed in the UK in both the National Health Service (NHS) and private sectors [1]. After implantation, a foreign body reaction occurs which results in the formation of a fibrous capsule around the implant. This represents a benign physiological immune system-mediated process, and, while it typically does not result in significant clinical issues, it may undergo tissue fibrosis and capsular contracture in up to 74% of patients leading to significant pain and breast deformity [2]. Although implants do not increase the risk of conventional breast cancer, we are becoming increasingly aware of other serious complications arising from the implant capsule such as breast implant-associated anaplastic large cell lymphoma (BIA-ALCL), and squamous cell carcinoma (BIA-SCC) [3, 4]. Both are thought to arise from chronic inflammation within the periprosthetic capsule. In BIA-ALCL, prolonged immune stimulation—potentially due to bacterial biofilm and mechanical irritation—leads to T-cell dysregulation and clonal expansion of CD30-positive cells, often involving mutations in the JAK/STAT pathway. BIA-SCC, on the other hand, is believed to result from chronic irritation causing squamous metaplasia of the capsule lining, followed by dysplasia and malignant transformation [5, 6].

Metastases to the breast from extramammary primary cancers are extremely rare, with reported rates of incidence ranging from 0.2% to ⁓2.7% [7]. The most common primary tumors that can metastasize to the breast are *lymphomas, melanomas, rhabdomyosarcomas, ovarian, and lung cancers.* To our knowledge, there have been no reports of metastasis to the breast from a lung primary in patients with breast implants. Thus, a possible link between the implant chronic pro-inflammatory dysregulation and an enhanced metastatic pattern has not yet been explored.

In this article, we describe two patients, both with breast implants, who primarily presented symptomatically through the breast clinic, and were subsequently diagnosed with metastasis to the breast from a lung primary.

## Case 1

**Figure 1:**
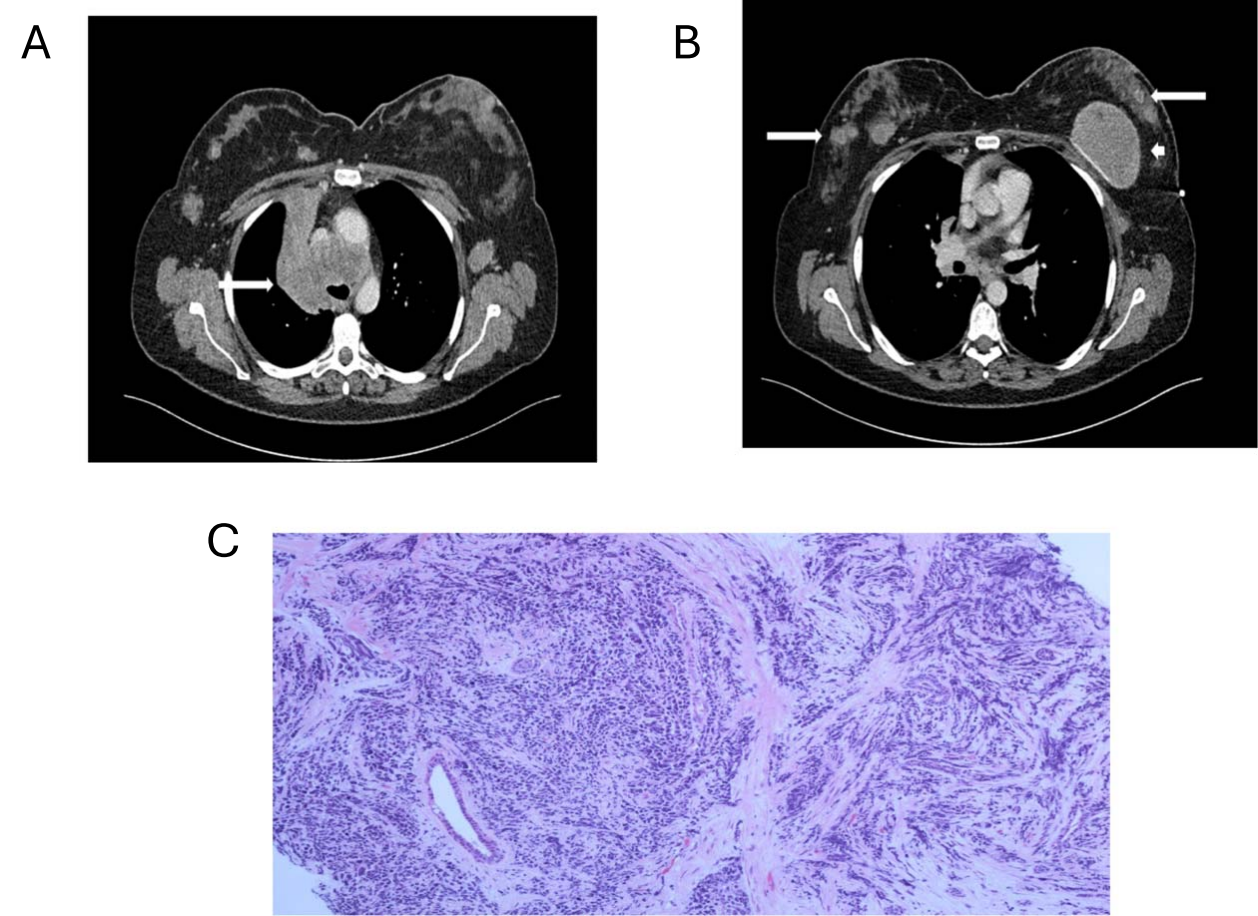
(A) Axial contrast enhanced CT shows right hilar mass. (B) CT scan showing bilateral breast metastasis (arrow) and left breast implant (arrowhead). (C) Cores of breast tissue infiltrated by malignant cells with high nuclear: cytoplasmic ratio, inconspicuous nucleoli and nuclear molding. The appearances are of small cell carcinoma.

**Figure 2:**
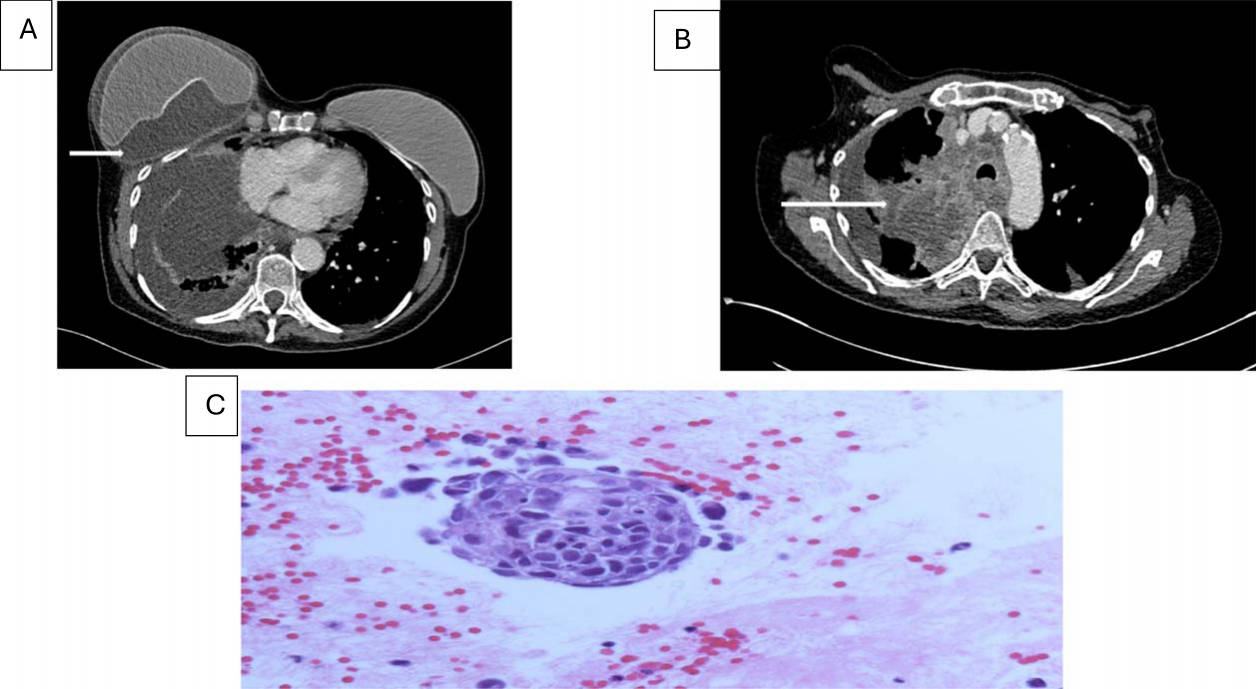
(A) Axial contrast enhanced CT scan shows heterogeneous lung mass with mediastinal invasion. (B) Axial CT image showing peri-implant malignant effusion. (C) fluid around breast implant: groups of atypical cells with hyperchromatic nuclei, high nuclear: cytoplasmic ratio and prominent nucleoli. The appearances are similar to the lung carcinoma diagnosed.

A 46-year-old female with a 20-year history of left breast augmentation performed for asymmetry, and a significant smoking history (55 pack-years), presented with a well-defined palpable lump in the upper outer quadrant of her right breast. On clinical examination she had bilateral breast lumps, initially suspected to be benign. Mammography and ultrasound imaging confirmed multiple bilateral breast masses, as well as an intracapsular rupture of the left breast implant (Fig. 1). The masses were classified as M2 (benign) on mammogram and U3 (indeterminate) on ultrasound, with an enlarged left axillary lymph node also identified. Core needle biopsies from the left breast mass and left axillary lymph node revealed metastatic involvement from a primary small cell carcinoma of the lung. Further staging with CT imaging revealed a contralateral right-sided lung tumor, along with extensive metastases to the hilar and mediastinal lymph nodes, liver, and pancreas (Fig. 2).

Despite the initiation of chemotherapy, the patient developed brain metastases and tragically passed away 8 months after the initial diagnosis.

## Case 2

A 73-year-old female, ex-smoker, with a history of bilateral breast cancer and delayed implant-based breast reconstruction (14 years and 10 years prior, respectively) presented with new swelling in the right breast. Clinical assessment, followed by ultrasound, revealed no evidence of implant rupture but identified a large seroma. The seroma was aspirated, and the fluid was sent for cytological analysis to exclude BIA-ALCL. The cytology report returned as inflammatory fluid.

Concurrently she was being investigated for cough and weight loss with a CT scan which noted a right-sided pleural effusion and further fluid accumulation around the breast implant, as well as a right upper lobe lung lesion (Fig. 2). A biopsy of the lung lesion confirmed a poorly differentiated squamous cell carcinoma and further aspirations of the persistent right breast peri-prosthetic effusion revealed metastatic malignant cells consistent with a primary lung origin.

The patient was initiated on chemotherapy; however, despite treatment, the lung lesion progressed. Unfortunately, the patient passed away 9 months after the initial diagnosis.

## Discussion

Metastasis to breast is a rare occurrence and even rarer from a lung primary [7]. Routes of metastatic spread may be via either a lymphatic or haematologic route with the former commonly presenting with an isolated, well-circumscribed mass as depicted in case 1. There have only been 61 cases of lung metastases to the breast reported in literature between 1989 and 2017 [8]. These cases are exceedingly rare, and notably, none of the patients involved in these reports had breast implants in situ.

The chronic inflammation induced by breast implants is one factor that may facilitate the development or progression of metastatic disease in susceptible individuals. Chronic inflammation has long been recognised as a key factor in tumorigenesis and metastasis. In fact, inflammatory responses are thought to create an environment that supports tumour growth, alters immune surveillance, and enhances angiogenesis [9]. This is especially relevant in the context of breast implants, as the body’s immune response to foreign materials can create sustained low-level inflammation around the implant site mediated by CD4 cells, potentially altering the tissue dynamics in a way that encourages tumor cell survival and dissemination.

A review of similar case reports in literature highlighted a relationship between the laterality of the primary lung cancer and metastasis, with most cases showing ipsilateral breast metastases [10]. Between 2013 and 2017, of the 18 cases identified, 13 were ipsilateral breast metastasis (72.2%). Studies have proposed that malignant lung cells may seed on the parietal pleura, invade the axillary lymphatic system and disseminate to the ipsilateral breast [8]. Other suggestions have included lymphatic drainage from mediastinal lymph nodes via supraclavicular or intercostal vessels may have led to the development of their ipsilateral axillary metastases. Despite documentation on ipsilateral metastasis, there remains a sparsity of literature on contralateral metastases as described in one of our cases.

These cases contribute to a narrow body of literature on lung cancer metastasis to the breast, in the unexplored setting of breast implant patient. Thus highlighting the complexities surrounding a possible connection between malignant diseases and the chronic inflammation associated with foreign body response and periprosthetic capsule formation.

## Conclusion

The rise in the use of breast implants has led to an increased awareness of the potential complications associated with these devices, especially since malignancies directly arising from the implant capsule (such BIA-ALCL and BIA-SCC) have been recently identified. However, metastatic disease involving the breast in patients with breast implants remains a rare and unreported phenomenon. This case series contributes to the literature by presenting two unique cases of lung cancer metastatic to the breast in patients with breast implants, both of whom primarily presented with breast-related symptoms. This highlights the potential for the peri-prosthetic environment to attract tumour cells or contribute to a pro-tumorigenic pathway. There is currently no strong evidence of a causative link between silicone implants and metastatic spread. Thus, silicone implants should not be considered a contraindication in oncologic patients on this basis alone. Further research may elucidate the causative pathway and identify patients that would be best to avoid silicone implants.
